# Ca^2+^ Requirements for Long-Term Depression Are Frequency Sensitive in Purkinje Cells

**DOI:** 10.3389/fnmol.2018.00438

**Published:** 2018-12-04

**Authors:** C. G. Zamora Chimal, Erik De Schutter

**Affiliations:** ^1^Computational Neuroscience Unit, Okinawa Institute of Science and Technology, Okinawa, Japan; ^2^Department of Biomedical Sciences, University of Antwerp, Antwerp, Belgium

**Keywords:** calcium requirements, frequency sensitive, long-term depression, cerebellum, CaMKII, stochastic simulation, Purkinje cell, molecular modeling

## Abstract

Cerebellar long-term depression (LTD) is a form of synaptic plasticity dependent on postsynaptic Ca^2+^ changes. One fundamental question is how LTD is selectively induced by specific numbers of Ca^2+^ pulses and which are the frequency and duration of this train of pulses required for LTD induction. The molecular mechanism which leads the integration of postsynaptic Ca^2+^ pulses in the LTD signaling network has not been elucidated either. Recent publications have shown that Ca^2+^/calmodulin-dependent protein kinase II (CaMKII) is required for LTD induction. Additionally, protein kinase C (PKC), CaMKII, and MAPK play an important role to transduce the frequency of Ca^2+^ pulses into their enzymatic activity levels; however, it is still unknown which enzymes are involved in decoding Ca^2+^ pulses in LTD. We have extended a stochastic model of LTD by adding the molecular network regulating CaMKII activity and its activation. We solved this model with stochastic engine for pathway simulation to include the effect of biochemical noise in LTD. We systematically investigated the dependence of LTD induction on stimulus frequencies, and we found that LTD is selectively induced by a specific number of Ca^2+^ spikes at different frequencies. We observed that CaMKII is essential to induce LTD, and LTD is only weakly induced when its Thr286 phosphorylation site has been deleted. We found that CaMKII decodes the frequency of Ca^2+^ spikes into different amounts of kinase activity during LTD induction. In addition, PKC and ERK enzyme activity is highly sensitive to the frequency and the number of Ca^2+^ pulses and this sensitivity has an important effect on LTD activation. This research predicts the postsynaptic Ca^2+^ requirements to induce LTD using a typical synaptic activation sequence and explains how LTD is selectively induced by specific number of Ca^2+^ pulses at different frequencies.

## Introduction

Cerebellar long-term depression (LTD) is a form of synaptic plasticity involved in motor learning (Ito, 2001). It is characterized as a robust and persistent decrease in the synaptic transmission between parallel fibers (PF) and Purkinje cells (PCs) which is induced by paired stimulation by PF and CF (Ito and Kano, 1982). Many lines of evidence suggest that postsynaptic calcium increases are necessary to induce depression of synaptic strength (Sakurai, 1990; Kasono and Hirano, 1994; Eilers et al., 1997; Miyata et al., 2000; Tanaka et al., 2007).

Purkinje cell expresses molecular mechanisms to detect conjunctive PF and CF activation to generate a calcium signal. CF input generates a strong depolarization causing opening of voltage gated calcium channels (VGCCs) and calcium influx entry into the spine (Miyakawa et al., 1992). PF input increases the Ca^2+^ concentration in the Purkinje dendritic spine via AMPA receptor and metabotropic glutamate receptor pathways. It has been shown that PF and climbing fiber (CF) coactivation causes a supralinear Ca^2+^ response in mammalian PCs (Wang et al., 2000). The supralinear calcium response to conjunctive PF-CF inputs is caused by a positive feedback loop in which a Ca^2+^ elevation increases Ca^2+^ release trough the IP_3_R (Doi et al., 2005).

The LTD is expressed as a reduction in the number of synaptic AMPA receptors (AMPAR) and depends on a protein kinase C (PKC)-ERK-cPLA_2_ positive feedback loop and a mechanism of AMPAR trafficking (Antunes and De Schutter, 2012; Gallimore et al., 2016). Experimental results show that CaMKII is required for the LTD induction; LTD is entirely absent in CaMKII-/- knockout mice slices (Hansel et al., 2006). Additionally, theoretical and experimental works have shown that CaMKII is sensitive to the frequency of Ca^2+^ oscillations (De Koninck and Schulman, 1998; Dupont et al., 2003). The activation and autophosphorylation of CaMKII by Ca^2+^ and calmodulin (CaM) are thought to influence its ability to decode Ca^2+^ spikes. However, the molecular mechanism by which this sensitivity contributes to LTD is not fully understood.

CaMKII is notoriously difficult to simulate in detail because of its multi-subunit nature, which produces a combinatorial explosion in the number of states and chemical species that must be simulated. Several models of CaMKII activation have been developed, but most of them have avoided the mechanistic, detailed modeling of every chemical reaction (Dupont et al., 2003; Li and Holmes, 2018). Developing a more detailed biochemical model may be useful to cover all possible combinations for activation of CaMKII and to explore their effects in more complex systems.

Hepburn et al. (2017) recently updated a stochastic model of the LTD signaling network including a PKC-ERK-cPLA_2_ feedback loop, Raf-RKIP-MEK interactions, and AMPAR trafficking. We have extended this model by adding the molecular network regulating CaMKII activity and its activation. The CaMKII molecular network is based on a published model by Kawaguchi and Hirano (2013), and we have built a new four subunit CaMKII model for the activation and phosphorylation of CaMKII. We incorporated this model into the LTD molecular network developed by Hepburn et al. (2017). Other LTD models including the CaMKII pathway have been developed, but they have not included the intrinsic stochasticity of biochemical interactions (Kawaguchi and Hirano, 2013). This new model includes the noise in the signaling networks that plays an important role in cellular processes (Simpson et al., 2009).

Previous work has measured the Ca^2+^ input requirements to induce LTD in PCs using local uncaging of Ca^2+^. These experiments used a single and prolonged pulse, and varied the amount of Ca^2+^ by changing the peak light intensity to show the relationship between Ca^2+^ concentration and LTD amplitude (Tanaka et al., 2007). However, typical experimental protocols to induce LTD use repeated synaptic activation, leading to multiple postsynaptic Ca^2+^ pulses. A fundamental question is how many pulses of Ca^2+^ are needed to induce LTD, and what is the optimal frequency and duration of this train of pulses for cerebellar LTD induction? This is useful to understand the postsynaptic Ca^2+^ requirements to induce LTD by a typical synaptic activation protocol and it is important to explain how LTD is selectively induced by a specific number of Ca^2+^ pulses.

Furthermore, the molecular mechanism that leads to the integration of this postsynaptic Ca^2+^ pulses in the LTD molecular network has not been elucidated. Regarding this, some studies have reported that PKC, CaMKII, MAPK play an important role to transduce the frequency of Ca^2+^ pulses into their enzymatic activity levels (Oancea and Meyer, 1998; Smedler and Uhlen, 2014); however, it is still unknown which kinases and phosphatases are involved in decoding the repeated Ca^2+^ pulses in the cerebellar LTD. The cerebellar LTD molecular network includes CaMKII, PKC, or MAPK like possible candidates to transduce the train of calcium pulses into the enzymatic activity required to induce LTD.

## Materials and Methods

We have extended a stochastic model of the LTD molecular network developed by Hepburn et al. (2017) by adding a molecular model of CaMKII activation and its regulating network.

### CaMKII Model

It has been shown that the CaMKII holoenzyme plays a critical role in decoding Ca^2+^ signals. Elucidating how this occurs has been under experimental and theoretical study (De Koninck and Schulman, 1998; Dupont et al., 2003; Li and Holmes, 2018). However, CaMKII is notoriously difficult to simulate due to its multi-subunit nature and the resulting combinatorial explosion in the number of states and chemical species that must be modeled. Researchers have developed phenomenological and biophysical models of CaMKII to understand the properties of this kinase. Some models of CaMKII activation have been created to avoid the detailed description of the biochemical reaction system of activation by CaM and its autophosphorylation (Hanson et al., 1994; Michelson and Schulman, 1994; Dosemeci and Albers, 1996; Li and Holmes, 2018) and more detailed models have been reported (Kubota and Bower, 2001; Chao et al., 2011).

**FIGURE 1:**
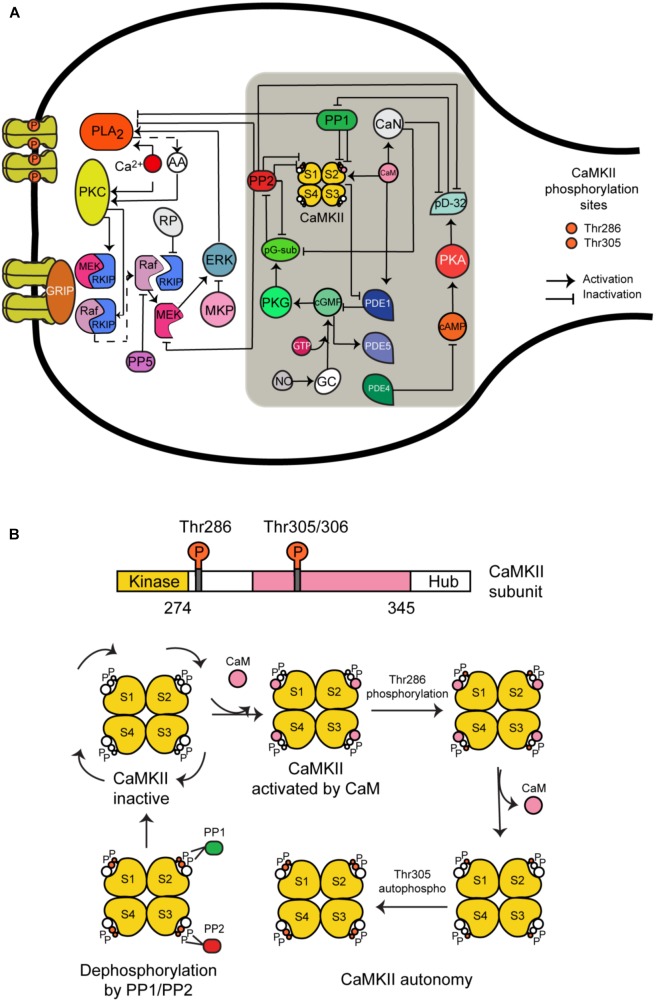
The long-term depression signaling pathway including the molecular network regulating CaMKII activity at postsynaptic density. **(A)** The molecules we have added to this signaling pathway are CaMKII (four subunits model); calmodulin (CaM), calcineurin (CaN); phosphodiesterases PDE1, PDE4, and PDE5; phosphatases PP1 and PP2A; protein kinase A (PKA) and G (PKG); cAMP and cGMP; NO; and DARP32. This figure was based on the previous model of LTD and extended with CaMKII molecular interactions (gray box). **(B)** All possible states of CaMKII activation.

CaMKII is a serine/threonine-specific protein kinase that is regulated by the Ca^2+^/CaM complex. It comprises 12 subunits, each of which contains a catalytic domain, a regulatory domain, and a carboxyl-terminal association domain. Every subunit can take one of five different states (Figure 1B):

(1) CaMKII inactive.
(2) CaM is bound to CaMKII and CaMKII becomes active.
(3) Phosphorylation at Thr286 when CaM is bound to a subunit.
(4) CaM is unbound from autophosphorylated subunit.
(5) Second phosphorylation at Thr305.

We developed a detailed molecular model of CaMKII activation and its dephosphorylation by PP1 and PP2. It is based on a previous model developed by Kubota and Bower (2001) that contains the same five states mentioned above. However, we added the dephosphorylation reactions at Thr286 and Thr305 phosphorylated sites by phosphatase 1 (PP1) and 2A (PP2A). First, we modeled CaMKII alone and calculated the number of possible states for four subunits when every subunit can take the five different states mentioned above. We included in our model 165 different states for activation of CaMKII calculated by the Necklace function (Li and Holmes, 2018), and we added the possible states following dephosphorylation reactions by PP1 and PP2A, making in total around 400 different states.

We have chosen PP1 and PP2A as phosphatases in our model based on previous experimental information that PP1 enhances the apparent cooperativity for Thr286 autophosphorylation of CaMKII (Bradshaw et al., 2003) and dephosphorylation of soluble and postsynaptic density CaMKII was catalyzed predominantly by PP2A and PP1 (Strack et al., 1997). Some experiments suggest that PP2A suppression recovered LTD under CaMKII suppression but this doesn’t happen when PP1 and PP2B (calcineurin) are suppressed (Kawaguchi and Hirano, 2013). Furthermore, several models of CaMKII phosphorylation and its dephosphorylation by PP1 and/or PP2A have been developed (Kubota and Bower, 2001; Kitagawa et al., 2009; Kawaguchi and Hirano, 2013).

The reactions included in the CaMKII model are: activation of the enzyme by Ca^2+^/CaM binding, intersubunit autophosphorylation at threonine residue Thr286, Ca^2+^-independent activation state through autophosphorylation and secondary intersubunit autophosphorylation at threonine residue Thr305/306.

### CaMKII Regulatory Molecular Network in LTD Signaling Pathway

Hepburn et al. (2017) recently updated a stochastic model of the LTD signaling network including a PKC-ERK-cPLA2 feedback loop, Raf-RKIP-MEK interactions, and AMPAR trafficking. We have extended this model by adding the molecular network regulating CaMKII activity and its activation (Figure 1A). Some of the most important new components of this network include CaMKII, phosphodiesterase 1 (PDE1), cGMP/protein kinase G (PKG), and nitric oxide (NO) pathway. These new components are based on a published model by Kawaguchi and Hirano (Kawaguchi and Hirano, 2013). A detailed description of the model can be found in the Supplementary Tables S1, S2. All model species and parameters are listed in Supplementary Table S1 and all model chemical reactions are listed in Supplementary Table S2.

### Simulations and Analysis

Noise in the signaling networks plays an important role in cellular processes (Simpson et al., 2009). We have simulated the LTD molecular network using the well-mixed solver of Stochastic Engine for Pathway Simulator Hepburn et al. (2012) to simulate the influence of noise on the LTD signaling network.

We computed the mean response of the LTD model over 100 iterations with different random seeds. The LTD response was calculated as the proportion of synaptic AMPARs 30 min after a Ca^2+^ input stimulus at the starting time of the simulation. The input calcium signal was designed as a consecutive train of pulses, each with a duration of 200 ms (except for 10 Hz, 80 ms) and an amplitude of 1.8 μM on average and 0.18 μM of SD. We calculated the response of the system for every 10 number of calcium pulses, from 0 to 100 pulses at frequencies of 0.1, 1, 4, and 10 Hz.

We calculated the probability to get LTD at a specific number of calcium pulses for every frequency. LTD was considered successful when a single trial reached the equilibrium state with the mean of AMPA receptors in the last 3 min of the simulation less than the 70% from the initial value. We counted the number of successful events over the total of simulation experiments (100 trials). LTD probability values range from 0 to 1, the maximum probability is 1 when all the trials showed LTD. The curves are fitted by Hill equation, as follows:

$$LTD=LTD_{\max}\frac{Ca_{\mathrm{pulses}}^{2+}\mathrm{nHill}}{K_{1/2}^{\mathrm{nHill}}+Ca_{\mathrm{pulses}}^{2+}\mathrm{nHill}}$$

Sigmoidal curves were obtained by the Curve fitting toolbox of Origin (Origin 8, Microcal, Northampton, MA, United States). LTD means the probability to get LTD over 100 trials. The parameters of the curves are Hill coefficient (nHill), the number of Ca^2+^ pulses required to achieve the half maximum magnitude of LTD (K_1/2_), and the maximum probability LTD_max_.

We changed the width of calcium pulses to 90 ms and 500 ms at 1 and 0.1 Hz to show the effect of stimulation width on LTD probability, and we changed the amplitude of calcium influx per pulse to 4–5 μM and 10–11 μM at 1 Hz to emulate a supralinear increase of calcium influx and its effect on LTD probability.

## Results

Through stochastic modeling we built a detailed mechanistic model of CaMKII activation and we included this model into the LTD molecular network in order to study the postsynaptic Ca^2+^ requirements to induce LTD using a typical synaptic activation procedure (train of calcium pulses) and to explain how LTD is selectively induced by specific numbers of Ca^2+^ pulses. In addition, we studied which kinases are involved in transducing the repeated Ca^2+^ pulses into their enzymatic activity in the induction of cerebellar LTD.

### The LTD Signaling Network Is Bistable

**FIGURE 2:**
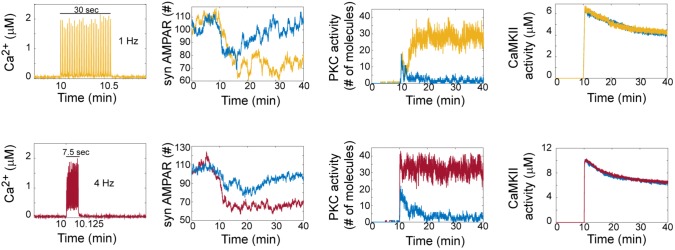
LTD signaling network is bistable. Molecular network activity over time with thirty Ca^2+^ pulses as stimulus at 10 min at frequecies of 1 Hz and 4 Hz. Ca^2+^, AMPAR, PKC, and CaMKII activity over time at 1 Hz (yellow train of Ca^2+^ pulses) and 4 Hz (red train of Ca^2+^ pulses). Blue color represents the dynamic of non LTD response.

Hepburn et al. (2017) reported in a previous model that LTD is bistable. In our model, we clearly observed that AMPAR trajectories can be in two different states (LTD or non LTD). An LTD response is defined as a reduction in synaptic AMPARs of around 30%, while non-LTD occurs when it is around 0 % (Figure 2). At zero calcium pulses input, all trials of the model are non-LTD responses and this response gradually changes to LTD when the number of calcium pulses increases. Interestingly, increasing the number of calcium pulses affects the probability of LTD induction and shows the coexistence of LTD and no LTD states at low number of calcium pulses.

To understand how bistability arises in the LTD molecular network, we analyzed the activation of PKC and CaMKII in single trials in the model at 1 and 4 Hz (Figure 2). For LTD responses, we clearly observe that LTD and non LTD states are related with the activation of PKC for both frequencies, and this PKC activity over time shows bistability. CaMKII activity reaches its maximum activity and is dephosphorylated over time for LTD and non LTD. Interestingly, in comparison with the state of non-LTD (blue color) CaMKII shows little difference in activity when the LTD is active (yellow and red color), decreasing monotonically after 30 min for both frequencies.

**FIGURE 3:**
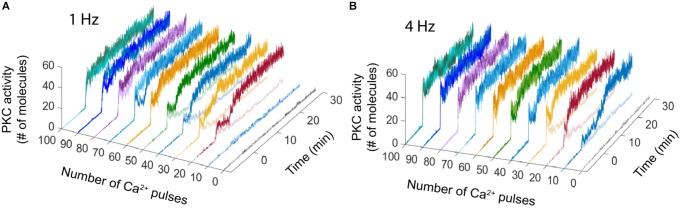
PKC activity over time shows bistability. Raw data of PKC over time in the LTD signaling network at different number of Ca^2+^ pulses. Two different trials were chosen randomly for every number of Ca^2+^ input. **(A)** Bistability of PKC activity is clearly visible from 20 to 50 calcium pulses at 1 Hz and **(B)** from 10 to 30 pulses at 4 Hz.

The bistable dynamic of AMPAR and PKC is less common when the number of calcium pulses increases, high number of Ca^2+^ pulses reduces the number of failures of LTD induction and PKC activation. We analyzed the bistability in PKC activity over time at 1 and 4 Hz. PKC activation is bistable when the stimulus is a low number of Ca^2+^ pulses, for 1 Hz we observe bistability from 20 to 50 pulses and for 4 Hz from 10 to 30 pulses (Figure 3). For both frequencies, PKC activity shows either a non-active state, delayed activation or full activation in the bistability region, with full activation of PKC becoming most common when the number of calcium pulses are high (Figures 3A,B).

Experimental studies show that CaMKII is essential to induce cerebellar LTD (Hansel et al., 2006). We observed that CaMKII is active once Ca^2+^ input is induced in the system and its activity level is changed by the number of Ca^2+^ pulses, its amplitude and frequency (Figure 2). In our model, when CaMKII is absent LTD is not induced which is congruent with experimental results (Hansel et al., 2006). We found that for LTD or non-LTD states PKC leads the induction of LTD and CaMKII remains active for both states.

The contribution of CaMKII to LTD signaling pathway is independent of the positive feedback loop activity. When PKC is equal to zero and consequently LTD is inhibited, the activity and phosphorylation of CaMKII remain active. This shows that CaMKII enzyme is activated by Ca^2+^/CaM and its activity is independent of the positive feedback loop. However, CaMKII has an important role in the positive feedback loop as we describe below.

When CaMKII is not present in our system PKC activity is reduced to low levels and LTD is not activated, PKC is regulated through indirect inhibition by PP2A in the system. PDE1 activity is promoted when CaMKII is inhibited and this supresses the cGMP/protein PKG pathway, as a consecuence PP2A is controling and inhibiting the PKC feedback loop. These results are congruent with Kawaguchi and Hirano’s observations (Kawaguchi and Hirano, 2013).

### Minimum Ca^2+^ Pulses to Induce LTD

Long-term depression is induced by a transient rise in Ca^2+^ concentration in the postsynaptic PC (Kasono and Hirano, 1994; Finch et al., 2012). A fundamental question is to determine the minimum of Ca^2+^ pulses necessary to induce LTD and which are the frequency and duration of this train of pulses for cerebellar LTD induction. Karachot et al. (1994) reported the stimulus parameters for induction of LTD at PCs. They systematically investigated the dependence of LTD induction on stimulus frequencies and the number of stimuli. Tanaka et al. (2007) studied the Ca^2+^ input requirements to induce LTD at postsynaptic neurons using local uncaging of Ca^2+^. These experiments showed the relationship between Ca^2+^ amount and LTD using a single calcium pulse; however, under physiological conditions, LTD is induced by several calcium transients at PCs. To define the Ca^2+^ requirements for LTD using a typical synaptic activation, we investigated the dependence of LTD on the number of Ca^2+^ pulses at different frequencies. We stimulated the LTD signaling network with a train of Ca^2+^ pulses, from 0 to 100 pulses at 0.1, 1, 4, and 10 Hz to know the minimum requirements of Ca^2+^ to induce LTD.

**FIGURE 4:**
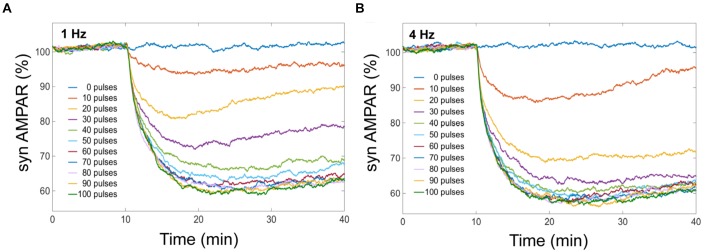
LTD is selectively induced by specific number of Ca^2+^ pulses. Mean of number of AMPA receptors when depression is induced by different number of Ca^2+^ pulses at different frequencies. Successful LTD is obtained when the mean of AMPA receptors decreased to less than 70%. **(A)** At 1 Hz the minimum number of calcium pulses to observe LTD is 50, and **(B)** LTD is induced by 30 pulses at 4 Hz.

We obtained from our model that averaged cerebellar LTD has different response levels, and this level is sensitive to the number of Ca^2+^ pulses (Figure 4). LTD at the postsynaptic density is selectively induced by specific number of Ca^2+^ spikes at different frequencies. When we have the same number of Ca^2+^ pulse stimuli but different frequency of calcium input, the amount of averaged LTD is quantitatively different. In our simulation experiments, successful LTD (70% or less on average) is induced after 50 pulses at 1 Hz and after 30 pulses at 4 Hz (Figure 4). At low number of calcium pulses LTD is not stable and with increasing number of pulses the LTD level increases and becomes stable.

### LTD Probability Over Number of Ca^2+^ Pulses

**FIGURE 5:**
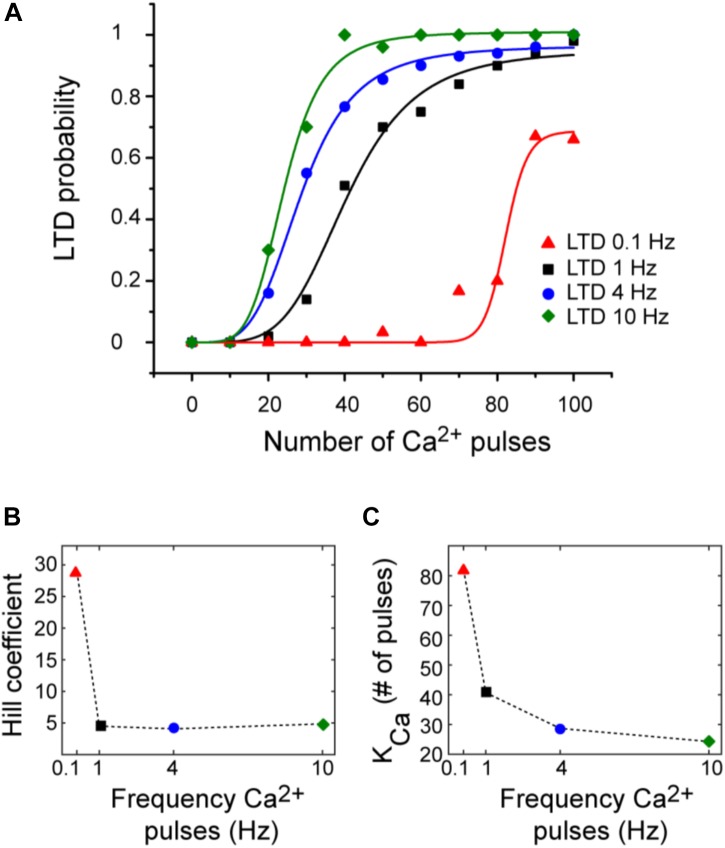
LTD probability over number of Ca^2+^ pulses at 0.1, 1, 4, and 10 Hz. **(A)** Relationship between number of Ca^2+^ pulses and LTD for different frequencies of 0.1 Hz (red), 1 Hz (black), 4 Hz (blue), and 10 Hz (green). Every symbol represents the probability to get LTD state at specific number of Ca^2+^ pulses and frequency over 100 trials. Smooth curves represent fits of the Hill equation. **(B)** nHill coefficient at the four different frequencies and **(C)** K_Ca_.

Tanaka et al. (2007) defined the relation between Ca^2+^ concentration and LTD amplitude in uncaging experiments. They obtained a sigmoidal relationship between Ca^2+^ pulse and LTD. However, typically protocols to induce LTD use repeated synaptic activation leading to postsynaptic Ca^2+^ pulses. To describe the relationship between number of Ca^2+^ pulses and LTD, we calculated the probability function over Ca^2+^ number pulses (Figure 5). These results indicate that Ca^2+^ sensitivity on LTD depends on the frequency of calcium input. We obtained a sigmoidal relationship between number of Ca^2+^ pulses and amount of LTD for each frequency. High number of calcium pulses caused a shift to the left in this relationship; this shift is because the K_ca_ is higher with low frequency. The Hill coefficient was around 5 and is comparable for all frequencies when the duration of calcium pulses is the same (200 ms). This dependency is congruent with the previous experimental results (Tanaka et al., 2007). LTD probability is increasing as a function of the number of calcium pulses and reaches faster the maximum LTD probability when the frequency of Ca^2+^ input is increased.

We can relate the bistability of PKC with the probability of LTD, the bistable PKC activity is less common when the probability is higher than 50% and PKC bistability is present when the LTD probability is less than the 50% (Figures 3, 5) or in a range of low calcium pulses number.

#### LTD Dependence on Calcium Influx Amplitude

Parallel fiber and CF coactivation causes large calcium signals in mammalian PCs (Wang et al., 2000). The calcium signals were much larger after paired stimulation of CF and PF inputs; and could reach peak values above 10 μM.

We carried out simulations to explore the effect of varying the calcium influx per pulse to observe the effect on the system’s bistability. We used two simulations experiments at 1 Hz. First, we changed the amplitude of Ca^2+^ signal from 2 μM to 4–5 μM, and then to 10–11 μM (Wang et al., 2000).

**FIGURE 6:**
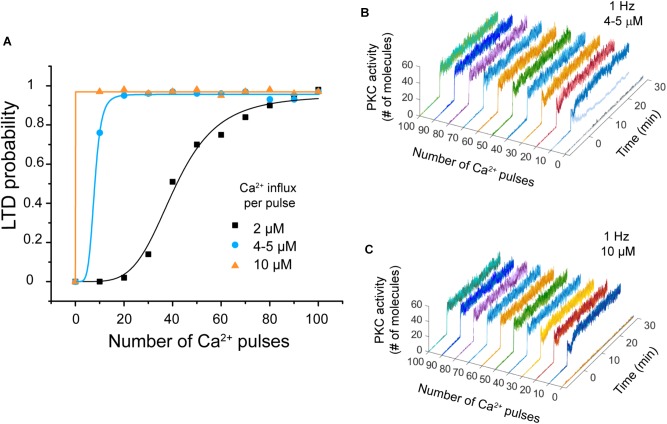
LTD probability as a function of calcium influx amplitude. **(A)** Relationship between number of Ca^2+^ pulses and LTD for different amplitudes of calcium influx per pulse at 1 Hz, 2 μM (black), 4–5 μM (blue), and 10–11 μM (orange). Every symbol (square, circle, or triangle) represents the probability to get LTD state at specific number of Ca^2+^ pulses and frequency over 100 trials. **(B)** PKC activity over time as a function of number of calcium pulses with an increment of amplitude to 4–5 μM. Bistability is only present at 10 pulses. (**C**) PKC activity as a function of number of calcium pulses with an increment of amplitude to 10 μM. PKC activity is always stable.

We observed that LTD probability increases when the calcium influx per pulse increases (Figure 6A). Regarding bistability, we observed that when the amplitude is increased to 4–5 μM PKC activity displays bistability at 10 Ca^2+^ pulses, but this bistability disappears if the amplitude is 10–11 μM (Figures 6B,C). LTD level increases and becomes stable when the calcium influx per pulse increases (10–11 μM) during PF-CF pairing.

#### LTD Dependence on Calcium Influx Width

**FIGURE 7:**
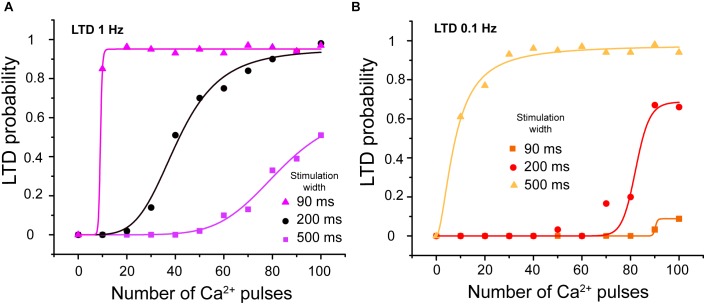
LTD probability as a function of calcium stimulus width. **(A)** Relationship between number of Ca^2+^ pulses and LTD for different widths of calcium influx per pulse, with 90, 200, and 500 ms at 1 Hz and **(B)** 0.1 Hz. Every symbol (square, circle or triangle) represents the probability to get LTD state at specific number of Ca^2+^ pulses and frequency over 100 trials. LTD probability increases when the width of calcium pulses increases and decreases when this decreases at both frequencies.

Calcium transients related to CF and PF recorded from PCs in cerebellar slices show that the calcium transients induced by paired PF and CF activation are around 200–300 ms of width (Wang et al., 2000). In our model, the calcium signal can be modulated by the peak amplitude, frequency, stimulation width, and duration of the train of pulses. We changed the width of calcium pulses to 90 or 500 ms at 1 and 0.1 Hz to show the effect of stimulation width on LTD probability. We observed that LTD probability increases when we increase the width of calcium pulses and decreases when we decrease it at the same frequency stimulation (Figures 7A,B).

The integrated calcium increases as we increase the stimulation width (results not shown) and consequently the LTD probability increases as a function of integrated calcium.

### Frequency Sensitivity in LTD Induction

**FIGURE 8:**
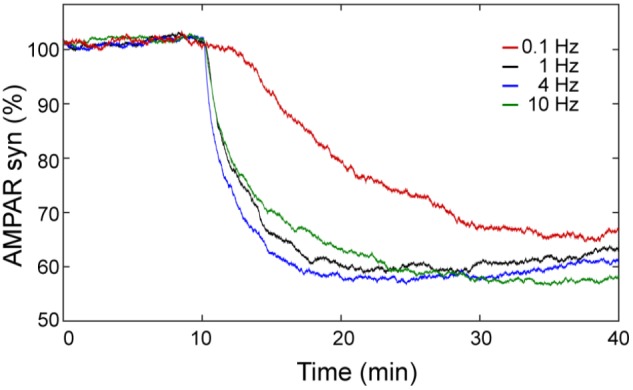
LTD is sensitive to the frequency of Ca^2+^ input signal. Mean over time of 100 trials of synaptic AMPAR with varying frequency of calcium pulses showing differences in the LTD amount. LTD was induced at 0.1 (red), 1 (black), 4 (blue), and 10 (green) Hz with the same Ca^2+^ input of one hundred of repetitive pulses (∼2 μM of amplitude) with a duration of every pulse equal to 200 ms.

The calcium sensitivity of LTD depends on the frequency of calcium input. In order to know the effect of the frequency of the calcium stimulus on LTD over time, we calculated the mean of 100 trials of AMPAR dynamic for frequencies at 0.1, 1, 4, and 10 Hz using 100 pulses of calcium (Figure 8). At this level of calcium stimulation, the LTD probability is almost 100% and most of the trials are stable LTD for most of the frequencies we have studied. However, we observed that for the same number of pulses the frequency of calcium signal plays an important role in the speed of LTD induction. At 0.1 Hz, the LTD induction is delayed in comparison with the cases at 1, 4, and 10 Hz and the reduction of AMPAR (70%) is only reached at the end of the simulation (40 min). At 1 and 4 Hz, successful LTD is reached after 20 min, and the levels of LTD are a little higher.

### Frequency Sensitivity in Signaling Network Model

**FIGURE 9:**
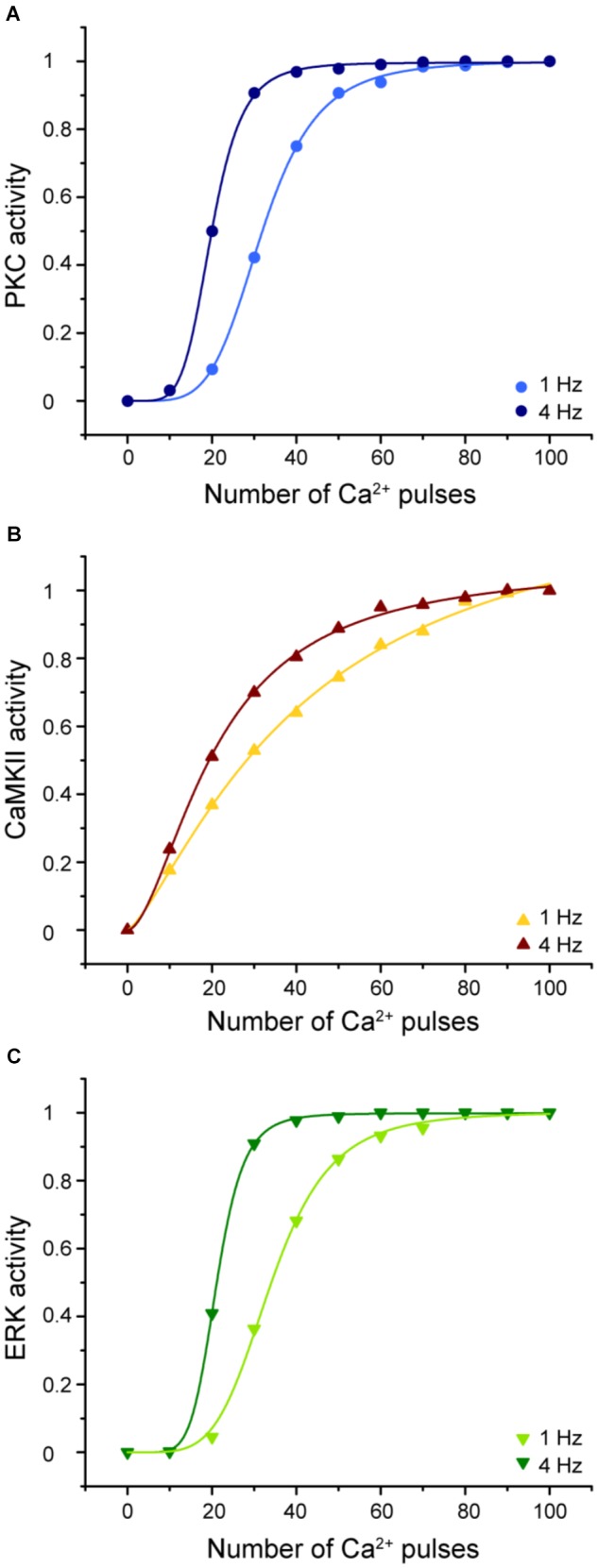
Enzymatic activity in the LTD signaling network is sensitive to the frequency and number of Ca^2+^ pulses. Sigmoidal relationship between the normalized activity of PKC, CaMKII, and ERK with number of calcium pulses at 1 and 4 Hz. **(A)** PKC, **(B)** CaMKII, and **(C)** ERK activity. Every symbol (dots for PKC, triangles for CaMKII and inverted triangles for ERK) represents the mean of 100 trials of enzyme activity at equilibrium state at 30 min after the calcium stimulus input.

Understanding the molecular mechanisms involved in synaptic plasticity, specifically how a postsynaptic calcium signal is transduced into a long-lasting change of synapses is an important question. To elucidate the molecular decoders of calcium signal into enzymatic activity in LTD molecular network we calculated the enzymatic activity of PKC, CaMKII, and ERK as a function of calcium pulses number at 1 and 4 Hz (Figure 9).

The activities of PKC, ERK, and CaMKII respond differently to stimuli with the same number of pulses, amplitude, and duration but varying frequencies. Increasing the stimulus frequency causes an increase in the activity of these enzymes. CaMKII decodes the frequency of Ca^2+^ spikes into different amounts of kinase activity during LTD induction. This result is congruent with previous studies of CaMKII sensitivity to Ca^2+^ oscillations (De Koninck and Schulman, 1998; Kubota and Bower, 2001). In addition, PKC and ERK activity is highly sensitive to the frequency, amplitude, and the number of Ca^2+^ pulses and consequently has an important effect on LTD activation. CaMKII, PKC, and ERK show a sigmoidal relationship between enzyme activity and calcium number pulses. These results indicate that the Ca^2+^ sensitivity of activity depends on the number of calcium pulses and its frequency and this suggest that these enzymes can integrate Ca^2+^ signals over time.

We already know that LTD induction has a dependence on number of calcium pulses and these Ca^2+^ requirements are related with the activation of kinases. PKC and ERK show a leftward shift of the dependence with increased stimulation frequency, while CAMKII shows mainly an increase in slope.

### CaMKII Activity in LTD Signaling Pathway

**FIGURE 10:**
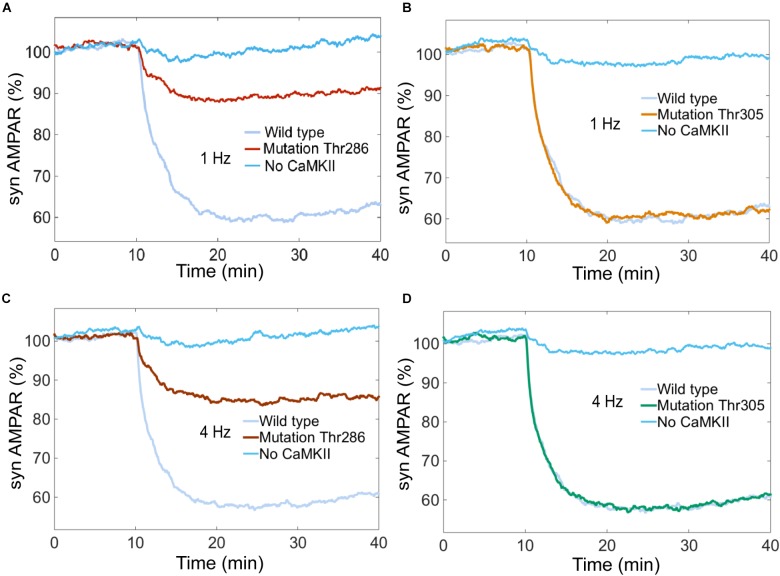
Thr286 phosphorylation site has an important effect on LTD induction. Time courses of depression induced by 100 of Ca^2+^ pulses at 1 and 4 Hz. Each curve shows the mean time course calculated for 100 iterations of the model. The magnitude of the depression was measured as percentage for synaptic AMPARs when the system is complete (wild type) and mutated at the Thr286 or Thr305 sites. **(A)** and **(C)** show LTD impairment when Thr286 phosphorylation site has been deleted for calcium stimulation at 1 and 4 Hz, respectively. **(B)** and **(D)** do not show any change in the amount of LTD when Thr305 phosphorylation site has been deleted. **(A–D)** show that CaMKII activity is required for LTD induction.

Experimental studies suggest that CaMKII is required for LTD induction (Hansel et al., 2006). We simulated the system without CaMKII with calcium stimulus at 0.1, 1, 4, and 10 Hz, and we found that LTD is not induced when CaMKII activity is null (Figures 10A–D).

CaMKII activation mechanisms include double phosphorylation at threonine sites Thr286 and Thr305. It is known that Thr286 phosphorylation site plays and important role for the sustained phosphorylation of CaMKII and Thr305 plays a role of negative feedback loop on CaMKII activation (Hanson and Schulman, 1992; Coultrap and Bayer, 2012). Pi et al. (2010) reported that CaMKII promotes either LTP or LTD, depending on the state of T305/T306 phosphorylation. Coultrap et al. (2014) found that LTD requires CaMKII Thr286 autophosphorylation. However, these findings were developed in hippocampus and the role of Thr286 and Thr305 phosphorylation sites in cerebellar LTD is unclear.

To know the effect of Thr286 and Thr305 phosphorylation sites on cerebellar LTD, we simulated a mutation of CaMKII at these phosphorylation sites. First, we calculated the AMPAR dynamics for two experiments, wild type and a mutation at Thr286 banning reactions at this site. The simulation experiment with the mutation at Thr286 has a negative effect on LTD, decreasing the probability to induce LTD after 30 min of simulation, the number of AMPAR over time decreased less than 10% after 30 min of stimulation by Ca^2+^ (Figures 10A,C). CaMKII is essential to induce LTD and reducing the sustained phosphorylation of CaM kinase II causes a decrement of about 30% on LTD induction. Second, we simulated a mutation of CaMKII at Thr305 site to know the effect on AMPAR dynamics. In this case, Thr286 phosphorylation is active meanwhile Thr305 is inactive, this mutation does not affect the induction of cerebellar LTD (Figures 10B,D).

## Discussion

We have modeled CaMKII activation and phosphorylation in a mechanistic way, explaining every reaction for all possible states for four subunits of CaMKII. We solved this model stochastically using stochastic engine for pathway simulation (STEPS). This CaMKII model may be useful to build other simulations of other systems, which require activation of CaMKII. We added this model to the LTD molecular network and we studied systematically the calcium requirements to induce LTD at postsynaptic density showing that cerebellar LTD is sensitive to the Ca^2+^ input frequency and selectively induced by specific number of Ca^2+^ pulses. This study provides a systematic understanding of Ca^2+^ stimulus parameters for LTD induction at the postsynaptic density.

Tanaka et al. (2007) studied the Ca^2+^ input requirements to induce LTD at postsynaptic neurons using local Ca^2+^ uncaging. These experiments showed a sigmoidal relationship between the amplitude of a single calcium pulse and LTD. In those experiments, the Ca^2+^ stimulus duration influences the calcium requirements for LTD induction, long elevation of calcium caused a leftward shift in the sigmoidal relationship. However, typically protocols to induce LTD use repeated synaptic activation leading to many postsynaptic Ca^2+^ pulses. We emulated the trains of synaptic activity typically used to induce LTD to explain how LTD is related to the number of Ca^2+^ pulses at the postsynaptic density. We found a sigmoidal relationship between the number of Ca^2+^ pulses and LTD amount with a similar relation as that in Tanaka et al. (2007); however, this sigmoidal function is changing as the stimuli frequency is increased. In our simulation experiments the Ca^2+^ stimulus frequency influences the calcium requirements for LTD induction. This dependency is closely related with the response of CaMKII, ERK, and PKC activity to the Ca^2+^ number pulses at specific frequencies.

Parallel fibers and CF coactivation causes supralinear calcium signals in PC (Wang et al., 2000). Supralinear calcium signals are generated by Ca^2+^-induced Ca^2+^ release via IP_3_R, and they can reach values above 10 μM in spiny dendrites (Wang et al., 2000). We showed that larger calcium signals may increase the LTD probability as a function of calcium pulses compared to a level of calcium around ∼2 μM (Figures 5, 6) based on Tanaka et al. (2007)). PKC bistability becomes stable as we increase the amplitude of calcium to 10 μM, this result would suggest that supralinear calcium signals may induce the LTD state for all the trials from the first pulses at 1 Hz. However, as there is a large discrepancy in the Ca^2+^ measurements between the Wang et al. (2000) and the Tanaka et al. (2007) studies, we consider this scenario unlikely.

Several theoretical and experimental works have studied the role of kinases to transduce calcium signals at specific frequency into their activity level. CaMKII has been studied as a decoder of Ca^2+^ signals (De Koninck and Schulman, 1998; Kubota and Bower, 2001; Dupont et al., 2003; Li and Holmes, 2018). Some studies have reported that PKC, CaMKII, and MAPK play an important role to transduce the frequency of Ca^2+^ pulses into their enzymatic activity levels (Oancea and Meyer, 1998; Smedler and Uhlen, 2014). Theoretical work has studied the correlation between MAPK cascade and periods of calcium oscillations (Yi et al., 2011). Kupzig et al. (2005) have studied experimentally the role of frequencies of Ca^2+^ oscillations on activation on Ras and the ERK/MAPK pathway, and it has been suggested that synaptic signaling network may function as a temporal decoder (Kubota and Bower, 1999; Bhalla, 2002). However, the relationship between enzymatic activity and postsynaptic Ca^2+^ pulses in cerebellar LTD has not been studied. The molecular network of LTD induction includes several molecular players that transduce Ca^2+^ spikes frequency into their enzymatic activity. CaMKII, PKC, and ERK enzyme activities are highly sensitive to the frequency and this is strongly related with the amount of LTD. PKC and ERK responses over calcium pulses are different when the stimuli are at different frequency. These responses are shifted to the left at 4 Hz in comparison with 1 Hz. Also, the slope of the sigmoidal of PKC and ERK changes with the frequency, at higher frequency the steepness is higher than at low frequency stimulation, this means that the enzyme reaches faster the maximum level of activity at 4 Hz than at 1 Hz. At the same frequency, the slope of ERK is more sensitive to the Ca^2+^ input because the enzyme activity reaches the 90% at 30 pulses, meanwhile PKC reaches the same activity at 40 pulses. This means that the threshold of ERK and PKC activation is different at specific number of Ca^2+^ pulses. CaMKII activity responses faster to Ca^2+^ input than ERK and PKC, its activity reaches around 20% when the enzyme is stimulated just by 10 pulses. However, the slope of the curve increases slowly for further increases in the number of Ca^2+^ pulses.

Hansel et al. (2006) examined the role of CaMKII in cerebellar plasticity using CaMKII-/- knockout mice. They showed by electrophysiological experiments that LTD was entirely absent 15–20 min after PF LTD induction in mutant slices. In contrast, slices obtained from wild type mice showed significant LTD. These results are congruent with our simulations experiments (Figure 10). However, there is no experimental information that shows the effect of specific CaMKII Thr286/305 phosphorylation sites on cerebellar LTD and its role to decode calcium transients at PCs. Our results are a target to be tested by electrophysiological experiments.

The importance of the Thr286 site on CaMKII activity has been studied extensively (Coultrap and Bayer, 2012; Lisman et al., 2012). Coultrap et al. found that LTD requires CaMKII and its phospho-T286-induced Ca^2+^-independent activity (Coultrap et al., 2014). Through stochastic modeling we have shown that the Thr286 phosphorylation site of CaMKII has a major effect on LTD induction, decreasing the amount of LTD when the Thr286 site has been deleted. Recent experiments have shown that CaMKII acts as a leaky integrator of Ca^2+^ pulses in dendritic spines and phosphorylation at Thr286 is necessary for optimal integration of Ca^2+^ pulses in hippocampal LTP (Chang et al., 2017). This is an important experimental observation, using our model we have shown that the Thr286 site is necessary to maintain LTD at different frequencies and we observed that CaMKII plays an important role to integrate the Ca^2+^ signal input. Further experimental studies may focus on cerebellar LTD to test the hypothesis about CaMKII threonine phosphorylation sites as an important integrator of Ca^2+^ pulses at different frequencies.

## Data Availability

The updated model of LTD signaling pathway is available at ModelDB: URL: http://senselab.med.yale.edu/ModelDB/showModel.cshtml?model=245412.

## Author Contributions

CZC contributed to the LTD model, ran simulations, analyzed data, and wrote the manuscript. EDS supervised the project and wrote the manuscript.

## Conflict of Interest Statement

The authors declare that the research was conducted in the absence of any commercial or financial relationships that could be construed as a potential conflict of interest.

## Supplementary Material

TABLE S1 Model parameters and chemical species.

TABLE S2 Model reactions.
